# Epigenetic silencing of miR-342-3p in B cell lymphoma and its impact on autophagy

**DOI:** 10.1186/s13148-020-00926-1

**Published:** 2020-10-19

**Authors:** Min Yue Zhang, George A. Calin, Kit San Yuen, Dong Yan Jin, Chor Sang Chim

**Affiliations:** 1 Division of Hematology, Renji Hospital, School of Medicine, Shanghai Jiaotong University, Shanghai, China; 2 Department of Medicine, Queen Mary Hospital, The University of Hong Kong, Pokfulam Road, Pokfulam, Hong Kong; 3 Translational Molecular Pathology Department, The University of Texas MD Anderson Cancer Center, Houston, TX USA; 4 School of Biomedical Sciences, The University of Hong Kong, Pokfulam Road, Pokfulam, Hong Kong

**Keywords:** Autophagy, B cell lymphoma, DNA methylation, miR-342-3p, Tumor suppressor miRNA

## Abstract

**Background:**

miR-342-3p, localized to 14q32, is a tumor suppressor miRNA implicated in carcinogenesis. Given the presence of a promotor-associated CpG island for its host gene, *EVL*, we hypothesized that intronic miR-342-3p is a tumor suppressor co-regulated with host gene by promoter DNA methylation in B cell lymphoma.

**Results:**

By bisulfite pyrosequencing-verified methylation-specific PCR (MSP), *EVL/MIR342* methylation was detected in five (50%) lymphoma cell lines but not normal peripheral blood and tonsils. *EVL/MIR342* methylation correlated with repression of both miR-342-3p and EVL in cell lines. In completely methylated SU-DHL-16 cells, 5-AzadC treatment resulted in promoter demethylation and re-expression of miR-342-3p and EVL. In 132 primary lymphoma samples, *EVL/MIR342* was preferentially methylated in B cell lymphomas (*N* = 68; 68.7%) than T cell lymphoma (*N* = 8; 24.2%) by MSP (*P* < 0.0001). Moreover, *EVL/MIR342* methylation was associated with lower miR-342-3p expression in 79 primary NHL (*P* = 0.0443). In SU-DHL-16 cells, the tumor suppressor function of miR-342-3p was demonstrated by the inhibition of cellular proliferation and increase of cell death upon over-expression of miR-342-3p. Mechanistically, overexpression of miR-342-3p resulted in a decrease of LC3-II, a biomarker of autophagy, which was pro-survival for SU-DHL-16. Pre-treatment with 3-methyladenine, an autophagy inhibitor, abrogated tumor suppression associated with miR-342-3p overexpression. By luciferase assay, MAP1LC3B, a precursor of LC3-II, was confirmed as a direct target of miR-342-3p. Finally, in SU-DHL-16 cells, overexpression of miR-342-3p downregulated the known target DNMT1, with promoter demethylation and re-expression of tumor suppressor E-cadherin.

**Conclusions:**

Intronic miR-342-3p is co-regulated with its host gene EVL by tumor-specific promoter DNA methylation in B cell lymphoma. The tumor suppressor function of miR-342-3p was mediated via inhibition of pro-survival autophagy by targeting MAP1LC3B and downregulation of DNMT1 with demethylation and re-expression of tumor suppressor genes.

## Background

Non-Hodgkin’s lymphoma (NHL) is the most common hematological malignancy. The incidence of NHL was 6.7/100,000 among males and 4.7/100,000 among females [1]. Based on the lineage and maturity of neoplastic cells, NHL can be classified as precursor and mature form of B-, T- or NK-cell lymphoma [2]. B-cell lymphoma, comprising > 70% of all NHLs [3], can be further classified into different subtypes based on morphology, immunophenotyping and genetic alterations. The most common subtype of B-cell lymphoma is diffuse large B-cell lymphoma (DLBCL), followed by follicular lymphoma (FL) whereas NK-cell lymphoma is an aggressive subtype rare in Western countries [4].

DNA methylation refers to the addition of a methyl group (-CH_3_) to carbon five of the cytosine ring in a CpG dinucleotide [5], catalyzed by DNA methyltransferases. Cancer cells are characterized by global DNA hypomethylation and locus-specific DNA hypermethylation of promoter-associated CpG islands of tumor suppressor genes (TSGs) [6], leading to reversible silencing of TSGs [5]. To date, promoter DNA methylation-mediated silence of TSGs, such as *p16*^*INK4a*^, *SOCS3* and *SHP1* [7–9], has been implicated in the pathogenesis of B-cell lymphoma.

microRNAs (miRNAs) are a class of single-stranded non-coding RNAs of 19~25 nucleotides in length [10]. Functionally, based on sequence complementarity between seed region of miRNA and seed region binding site on 3’-untranslated region (3’-UTR) of its corresponding target gene, the miRNA may downregulate the targeted mRNA through translational block or mRNA degradation [11, 12]. Dysregulated expression of miRNAs has been implicated in carcinogenesis [13]. Promoter DNA methylation has been shown to serve as an alternative mechanism leading to inactivation of tumor suppressor miRNAs, such as miR-129-2, miR-155-3p, miR-124-1 and miR-34a, in B-cell lymphoma [14–17].

*MIR342* is embedded in the third intron of its host gene *Enah/Vasp-like (EVL)* localized to 14q32. EVL, belonging to the Ena/VASP family of proteins, was reported to be a multifunctional regulator of actin cytoskeleton remodeling, actin polymerization and cell adhesion [18–20]. In glioblastoma and breast cancer, expression of EVL was higher in tumor tissues than normal tissue [21, 22]. Furthermore, the upregulation of EVL correlated with advanced stage of breast cancer, and promoted migration of MCF-7 breast cancer cells [21]. On the contrary, expression of EVL was found to be reduced in colorectal cancer and cervical cancer tissues compared with those in adjacent normal tissues [23, 24], hence a tissue-specific expression of EVL in different types of cancer. However, expression and biological function of EVL in lymphoma remains unknown. On the other hand, the tumor suppressor role of miR-342-3p via inhibition of cell proliferation, migration and invasion has been demonstrated in colon, lung, breast and hepatocellular carcinoma, by downregulation of oncogenic targets, including FOXQ1, DNMT1, MYC and IKK-γ [25–29]. However, little is known about its role in the pathogenesis of B-cell lymphoma. As a CpG island is present at the promoter of *MIR342* host gene *EVL*, we hypothesized that miR-342-3p is a tumor suppressor miRNA silenced by promoter DNA methylation of its host gene EVL in B-cell lymphoma. In the present study, promoter DNA hypermethylation of *EVL/MIR342* and the mechanism of tumor suppression of miR-342-3p were investigated in B-cell lymphoma.

## Results

### Methylation of *EVL/MIR342* in normal healthy controls and NHL cell lines

**Fig. 1:**
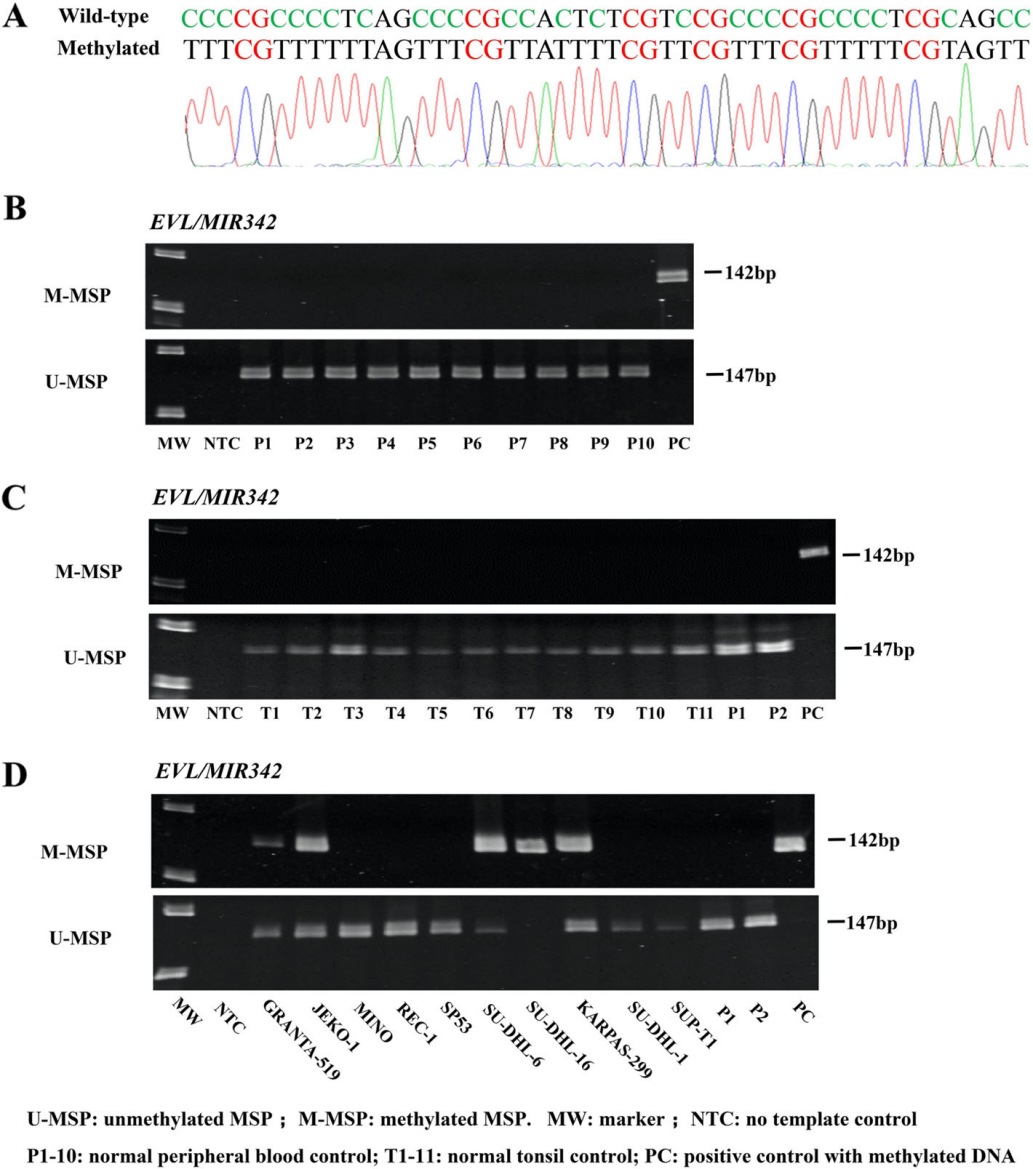
Methylation of *EVL/MIR342* in healthy normal controls and NHL cell lines. **a** Direct sequencing of M-MSP products from enzymatically methylated positive control DNA showed complete bisulfite conversion and MSP specificity. **b**, **c** Results of both M- and U-MSP showed absence of *EVL/MIR342* methylation in healthy normal controls, including normal peripheral blood buffy coats (P1-P10) (**b**) and normal tonsil tissues (T1–T11) (**c**). **d** In lymphoma cell lines, M- and U-MSP showed that *EVL/MIR342* was completely methylated (MM) in SU-DHL-16, partially methylated (MU) in JEKO-1, GRANTA-519, SU-DHL-6, and KARPAS-299, and completely unmethylated (UU) in MINO, REC-1, SP-53, SU-DHL-1, and SUP-T1

By methylation-specific PCR (MSP), promoter DNA methylation of *EVL/MIR342* was studied in the bisulfite-converted DNA of normal healthy controls, including peripheral blood buffy coats (*n* = 10) and tonsil tissues (*n* = 11), and NHL cell lines (*n* = 10). Direct sequencing of the M-MSP products amplified from an enzymatically methylated positive control DNA showed complete conversion of all unmethylated cytosines into thymidines after PCR, while all methylated cytosines in CpG dinucleotides remained unchanged, demonstrating complete bisulfite conversion and MSP specificity (Fig. 1a). MSP showed that *EVL/MIR342* was unmethylated in normal healthy controls (Fig. 1b, c). Conversely, in NHL cell lines, *EVL/MIR342* was completely methylated (MM) in SU-DHL-16, partially methylated (MU) in JEKO-1, GRANTA-519, SU-DHL-6, and KARPAS-299, and completely unmethylated (UU) in MINO, REC-1, SP-53, SU-DHL-1, and SUP-T1 (Fig. 1d). Furthermore, by quantitative bisulfite pyrosequencing, NHL cell lines showing MM, MU, and UU had a mean methylation level of 96.62, 44.20, and 5.30%, respectively (Additional file 1: Figure S1), confirming the methylation status derived by MSP. Taken together, *EVL/MIR342* was methylated in a tumor-specific manner in NHL cell lines.

### Methylation and expression of miR-342-3p and its host gene EVL in NHL cell lines

**Fig. 2:**
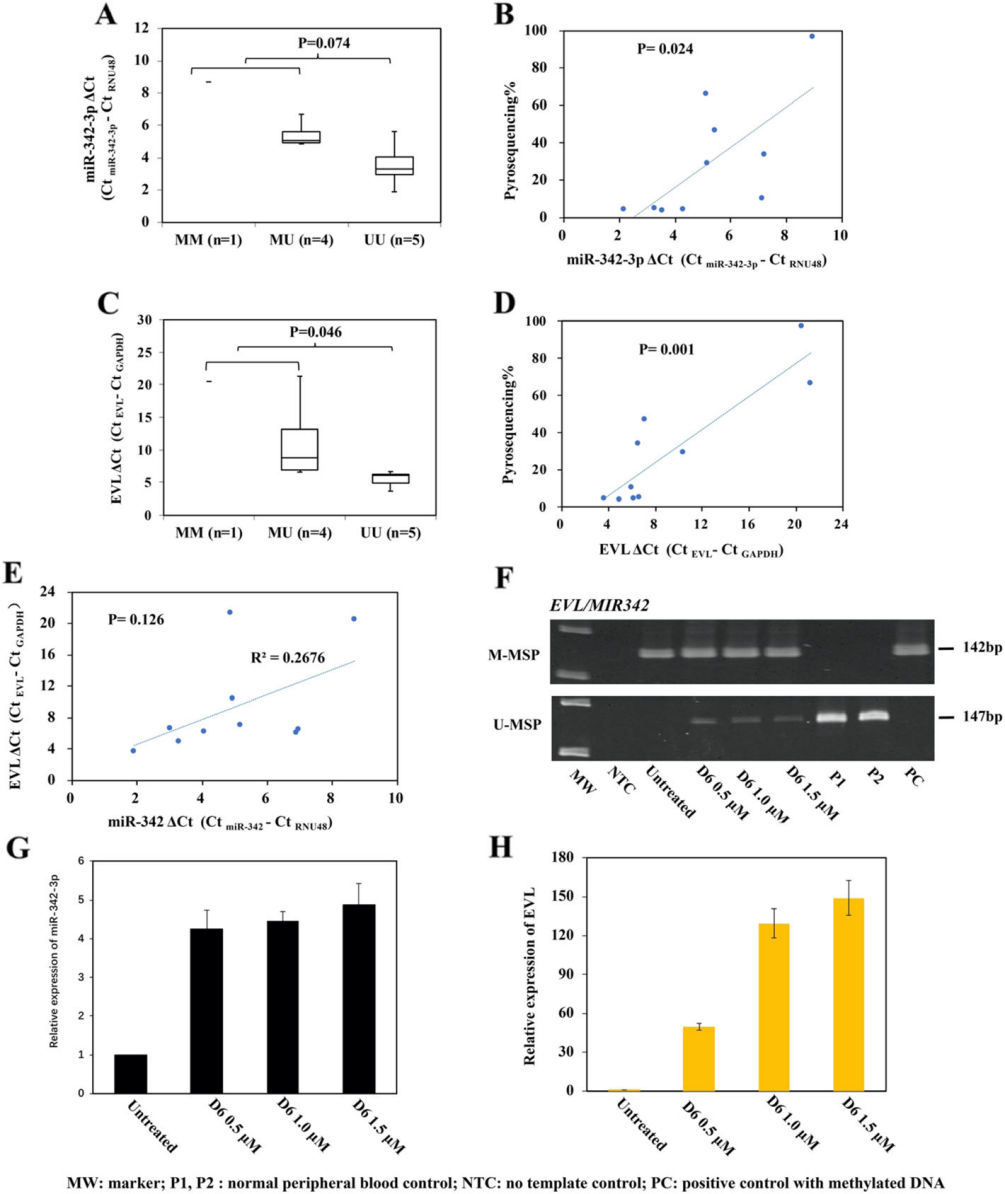
Methylation and expression of *EVL/MIR342* in NHL cell lines. **a**, **b** By qRT-PCR, the lower expression of miR-342-3p was associated with *EVL/MIR342* methylation as detected by MSP (**a**) and quantitative bisulfite pyrosequencing (**b**). **c**, **d** The lower expression of EVL, the host gene of miR-342-3p, was associated with *EVL/MIR342* methylation as measured by MSP (**c**) and quantitative bisulfite pyrosequencing (**d**). **e** By plotting the expression of miR-342-3p against its host gene EVL, a trend of concordant expression between miR-342-3p and its host gene EVL was demonstrated. **f**–**h** Treatment of SU-DHL-16 cells, completely methylated for *EVL/MIR342*, with 5-AzadC for 6 days led to *EVL/MIR342* promoter demethylation as shown by MSP (**f**), and re-expression of miR-342-3p (**g**) and EVL **(h)** as shown by qRT-PCR. qRT-PCR data of 5-AzadC treatment were normalized to untreated control as 1. Columns represented mean +/− 1SD from three qRT-PCR experiments in triplicate

To investigate the relationship between promoter DNA methylation of *EVL/MIR342*, and expression of miR-342-3p plus its host gene EVL, qRT-PCR of miR-342-3p and EVL were performed in NHL cell lines (*n* = 10). Results showed that lower expression of miR-342-3p was associated with *EVL/MIR342* methylation by MSP (MM + MU vs. UU, *P =* 0.074; Fig. 2a) or by quantitative bisulfite pyrosequencing (*P* = 0.024, Fig. 2b). Similarly, the lower expression of EVL was associated with *EVL/MIR342* methylation by MSP (MM + MU vs. UU, *P =* 0.046; Fig. 2c) or by quantitative bisulfite pyrosequencing (*P* = 0.001; Fig. 2d). Moreover, by plotting the expression of EVL against miR-342-3p, a trend of concordant expression between miR-342-3p and EVL was demonstrated (*R*^2^ = 0.27, *P* = 0.126; Fig. 2e); thereby, miR-342-3p was co-expressed with its host gene EVL in NHL cell lines.

To study if miR-342-3p and its host gene EVL were reversibly silenced by promoter hypermethylation, completely methylated SU-DHL-16 cells were treated with 5-aza-2′-deoxycytidine (5-AzadC), a demethylating agent. 5-AzadC treatment led to promoter demethylation of *EVL/MIR342*, as illustrated by the emergence of U-MSP signal on day 6 (Fig. 2f) and re-expression of miR-342-3p (Fig. 2g) and EVL (Fig. 2h), hence consistent with reversible silencing of miR-342-3p and its host gene EVL via promoter DNA methylation of *EVL/MIR342*.

### Methylation-mediated silencing of miR-342-3p in NHL primary samples

**Fig. 3:**
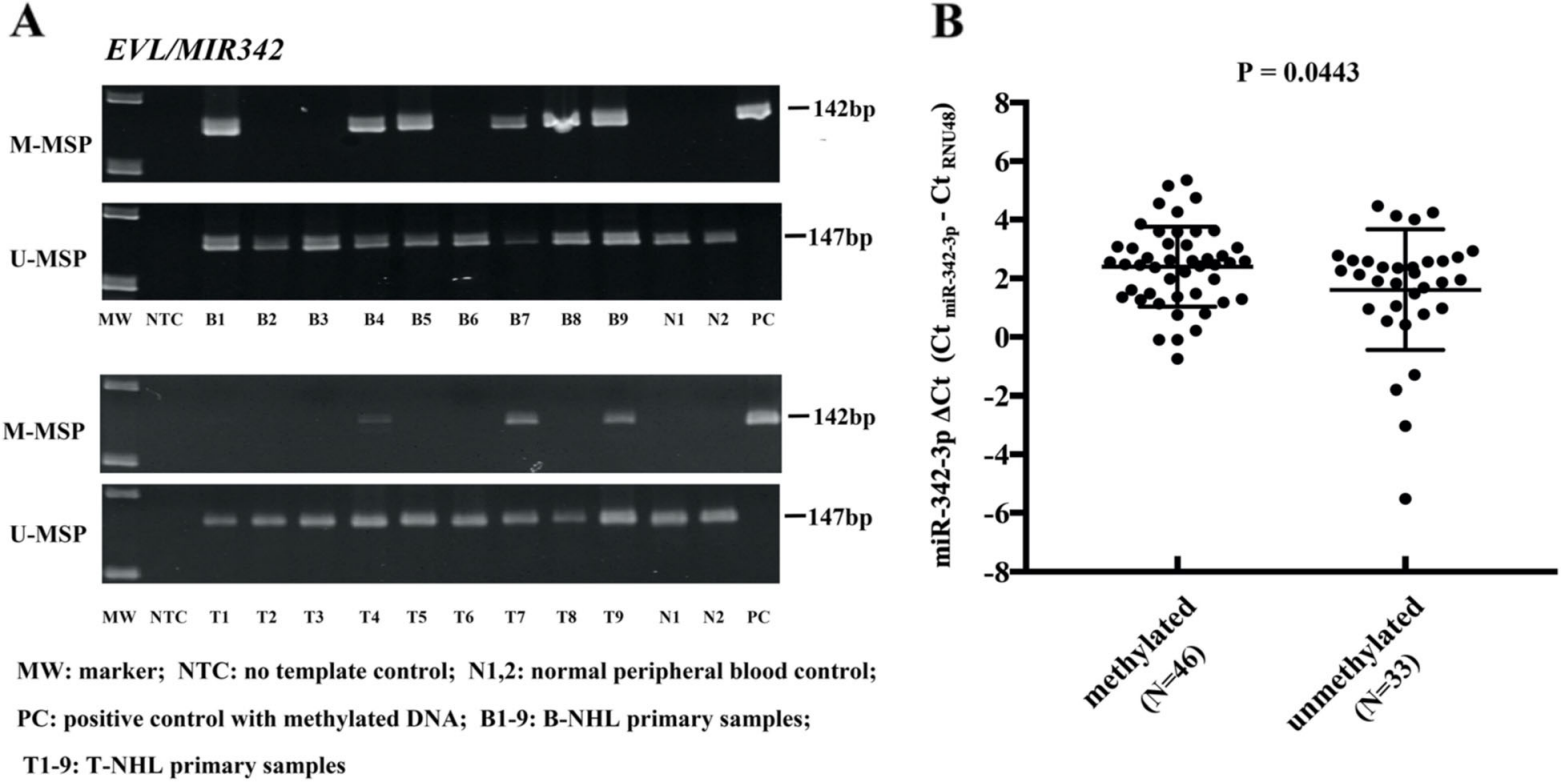
Methylation of *EVL/MIR342* and expression of miR-342-3p in primary NHL samples. **a** Representative M-MSP and U-MSP illustrated methylation of *EVL/MIR342* in primary samples of B cell lymphoma and T cell lymphoma. **b** In a total of 79 NHL patient samples, scatter plot analysis showed that the expression of miR-342-3p was significantly lower in patients with *EVL/MIR342* methylation than those without *EVL/MIR342* methylation

MSP was performed with bisulfite-converted DNA in 132 primary NHL samples. *EVL/MIR342* was found to be methylated in 68 (68.7%) B cell lymphoma and eight (24.2%) T cell lymphoma (Table 1, Fig. 3a), hence preferentially methylated in B cell lymphoma (*P* < 0.0001). The methylation frequency of *EVL/MIR342* among different subtypes of NHL was summarized in Table 1. In 57 patients with clinical data available, there was no association between *EVL/MIR342* methylation and overall survival (*P* = 0.606), age (*P* = 0.671), or sex (*P* = 0.576).

**Table 1:** *EVL/MIR342* methylation frequency in NHL primary samples

Disease subtype	Sample size	Methylation (%)
B-NHLs	99	68 (68.69%)
Diffuse large B cell lymphoma (DLBCL)	56	42 (75.00%)
Follicular lymphoma (FL)	6	4 (66.67%)
Mantle cell lymphoma (MCL)	17	4 (23.53%)
Burkitt’s lymphoma (BL)	5	4 (80.00%)
Mucosa-associated lymphoid tissue lymphoma (MALT)	4	4 (100.0%)
Marginal zone B cell lymphoma (MZBCL)	3	2 (66.67%)
Small lymphocytic lymphoma (SLL)	7	7 (100.00%)
Waldenstrom’s macroglobulinemia (WM)	1	1 (100.00%)
T-NHLs	33	8 (24.24%)
Peripheral T cell lymphoma (PTCL)	20	5 (25.00%)
Angioimmunoblastic T cell lymphoma (AITL)	13	3 (23.08%)

Based on qRT-PCR of miR-342-3p in 79 NHL primary samples, in whom both DNA and RNA were available, expression of miR-342-3p was shown significantly lower in patients with *EVL/MIR342* methylation than those without *EVL/MIR342* methylation (*P* = 0.0443, Fig. 3b).

### Effect of miR-342-3p overexpression and its impact on pro-survival autophagy in B cell lymphoma cells

**Fig. 4:**
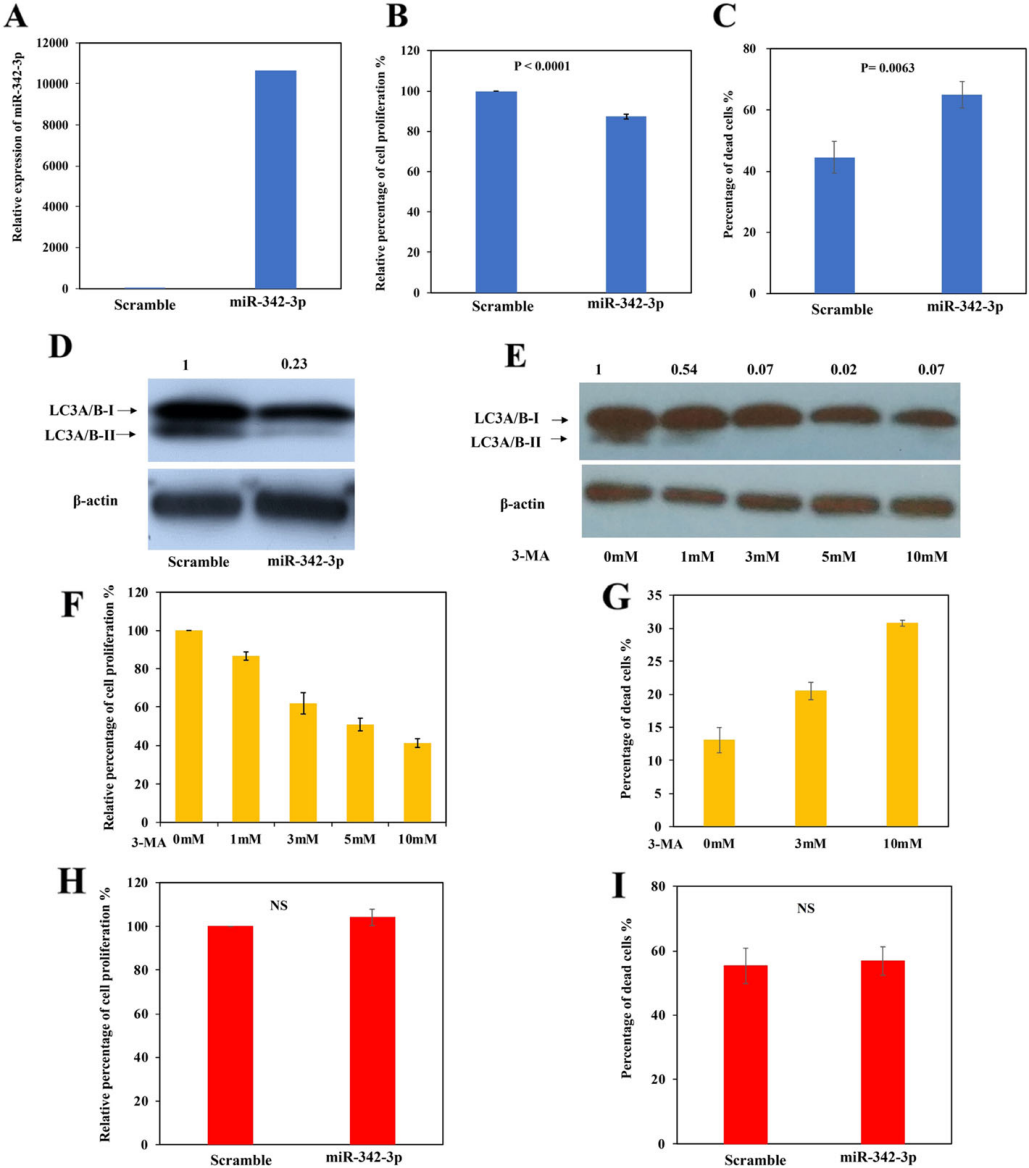
Tumor-suppressive function of miR-342-3p in SU-DHL-16 cells. **a** qRT-PCR analysis of miR-342-3p expression at 120 h after transfection. Data were normalized to negative scramble control as 1. **b** Cellular proliferation upon overexpression of miR-342-3p was analyzed by MTS assay. **c** Number of dead cells upon overexpression of miR-342-3p was calculated by trypan blue exclusion assay. **d** Western blotting analysis of LC3A/B protein with SU-DHL-16 cells transfected with miR-342-3p mimics or negative scramble control for 120 h. Numbers above the band represented the relative protein level of LC3A/B-II normalized to β-actin by densitometric analysis with Image J software. **e**–**g** SU-DHL-16 cells were exposed to a 24-h treatment with increasing concentrations of 3-methyladenine (3-MA), an inhibitor of autophagy, followed by (**e**) Western blotting analysis of LC3A/B protein, of which, LC3-II is a functional marker for autophagy, MTS assay (**f**) and trypan blue exclusion assay (**g**). Numbers above the band represented the relative protein level of LC3A/B-II normalized to β-actin by densitometric analysis with Image J software. **h**, **i** SU-DHL-16 cells were pre-treated with 3-MA (1 mM) for 24 h before transfection with miR-342-3p mimics or negative scramble control for 120 h, followed by MTS assay (**h**) and trypan blue exclusion assay (**i**). Data of MTS were normalized to negative scramble control or untreated control as 100%. Data of MTS assay were normalized to negative scramble control as 100%. Columns represent mean +/− 1SD from three experiments in triplicate

To investigate the biological role of miR-342-3p in B cell lymphoma cells, miR-342-3p mimics was transfected into SU-DHL-16 cells, which were completely methylated for *EVL/MIR342*. By qRT-PCR, successful overexpression of miR-342-3p was confirmed at day 5 after transfection (Fig. 4a). Moreover, as compared with negative scramble control, overexpression of miR-342-3p led to reduced cellular proliferation as measured by MTS assay (*P* < 0.0001, Fig. 4b), increased cell death by trypan blue exclusion assay (*P* = 0.0063, Fig. 4c), but no significant difference by annexin V-positive cells (figure not shown), and hence, these tumor-suppressive properties were mediated via mechanisms other than apoptosis.

Autophagy, an alternative mechanism for the regulation of cell survival [30], was studied for its role in the miR-342-3p-mediated tumor suppressive properties in B-cell lymphoma cells. Alteration of autophagy was monitored by Western blotting of LC3-II, which is a marker of autophagy. In completely methylated SU-DHL-16 cells, overexpression of miR-342-3p resulted in a reduction of LC3-II (Fig. 4d), indicating that miR-342-3p inhibited autophagy in SU-DHL-16 cells.

Moreover, the role of autophagy in the regulation of cellular proliferation and cell death was studied by treatment with an autophagy inhibitor, 3-methyladenine (3-MA), followed by Western blotting of LC3-II, MTS, and trypan blue assays. Upon treatment with 3-MA, autophagy in SU-DHL-16 cells was inhibited, as demonstrated by decreased expression of LC3-II by Western blotting (Fig. 4e). Moreover, 3-MA treatment resulted in decreased cellular proliferation by MTS assay (Fig. 4f) and increased cell death by trypan blue assay (Fig. 4g) in a dose-dependent manner, consistent with a pro-survival function of autophagy in SU-DHL-16 cells.

Furthermore, to confirm the role of pro-survival autophagy in miR-342-3p-mediated tumor-suppressive properties in B cell lymphoma cells, basal autophagy of SU-DHL-16 cells was first inhibited by pre-treatment with 3-MA prior to overexpression of miR-342-3p, followed by MTS and trypan blue analyses. Results showed that when basal autophagy was inhibited by pre-treatment with 3-MA, followed by overexpression of miR-342-3p, the tumor suppressor properties of miR-342-3p on inhibition of cellular proliferation (Fig. 4b) and induction of cell death (Fig. 4c) were completely abolished, as measured by MTS assay (Fig. 4h) and trypan blue exclusion assay (Fig. 4i), respectively, confirming that miR-342-3p exerted its tumor suppressor function via inhibition of pro-survival autophagy in B cell lymphoma cells.

### Identification of MAP1LC3B as a novel direct target of miR-342-3p

**Fig. 5:**
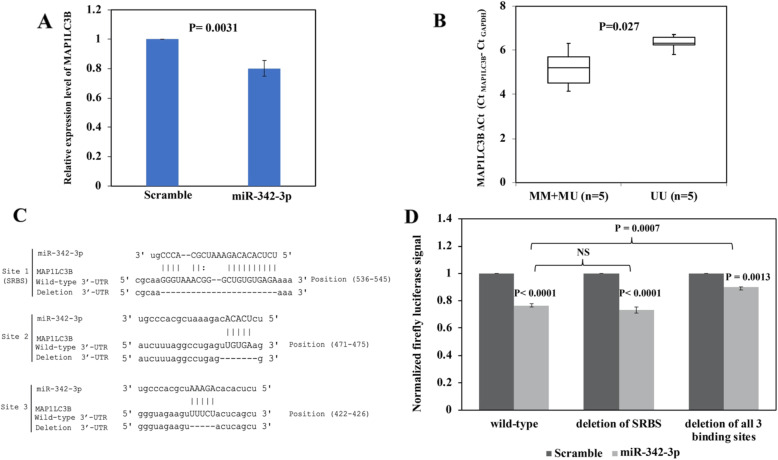
Identification of MAP1LC3B as a novel direct target for miR-342-3p. **a** By qRT-PCR, in SU-DHL-16 cells, the expression of MAP1LC3B was downregulated upon overexpression of miR-342-3p compared with negative scramble control. Data were normalized to negative scramble control as 1. **b** By qRT-PCR, the expression of MAP1LC3B was significantly higher in NHL cell lines methylated for *EVL/MIR342*. **c** Sequences of putative miR-342-3p binding sites in cloned fragment of MAP1LC3B 3′-UTR, showing one bioinformatically predicted putative seed region binding site (site 1) and two 5-mer binding sites (sites 2 and 3). The deletion mutant was generated according to the bottom line of each binding site. **d** Luciferase plasmids containing wild-type or mutant MAP1LC3B 3′-UTR were co-transfected with miR-342-3p mimics or scramble control into HeLa cells. Luciferase reporter assay was performed at 48 hours post-transfection. Co-transfection of both miR-342-3p mimics and wild-type MAP1LC3B 3′-UTR significantly reduced luciferase assay signals. Deletion of the putative SRBS did not restore the luciferase signal whereas deletion of all three binding sites could restore the luciferase activity as compared with wild-type MAP1LC3B 3′-UTR. Data were normalized to negative scramble control as 100%. Columns represented mean +/− 1SD from three experiments in triplicate

As miR-342-3p was shown to regulate autophagy, potential autophagy-related targets of miR-342-3p was studied. By miRWalk2.0 bioinformatic software [31], putative autophagy-related targets of miR-342-3p, matching to the 6-mer, 7mer-A1, 7mer-m8 and 8-mer miRNA seed sequences, included microtubule-associated proteins 1A/1B light chain 3B (MAP1LC3B), autophagy related 2B gene (ATG2B) and ATG10. Moreover, in completely methylated SU-DHL-16 cells, overexpression of miR-342-3p could result in downregulation of MAP1LC3B by qRT-PCR (*P* = 0.0031, Fig. 5a), but not ATG2B and ATG10 (data not shown). MAP1LC3B, which is a post-translational precursor form of LC3-II, a functional marker of autophagy, was further validated as a direct target of miR-342-3p.

By qRT-PCR, in 10 NHL cell lines, higher expression of MAP1LC3B was found in NHL lines with *EVL/MIR342* methylation (*P* = 0.027, Fig. 5b). Luciferase assay was employed to validate MAP1LC3B as a direct target of miR-342-3p. DNA fragment containing wild-type or deletion mutant seed region binding site (SRBS) (site 1; 8-mer; Fig. 5c) of the MAP1LC3B 3′-UTR were cloned into pmirGLO luciferase reporter. Upon co-transfection of both miR-342-3p mimics and wild-type MAP1LC3B 3′-UTR, the luciferase signal was significantly reduced compared with co-transfection with scramble control. However, when the putative SRBS was deleted, the luciferase signal was not restored (Fig. 5d), indicating the presence of other binding sites of miR-342-3p in the fragment of MAP1LC3B 3′-UTR downstream to the luciferase gene. Based on sequence complementarity analysis between miR-342-3p and the cloned fragment of MAP1LC3B 3′-UTR, two additional binding sites of miR-342-3p were identified (site 2 and 3; 5-mer; Fig. 5c); therefore, an additional deletion mutant truncating all three binding sites of the MAP1LC3B 3′-UTR was generated. Co-transfection of both miR-342-3p mimics and the mutant MAP1LC3B 3′-UTR with all binding sites deleted restored the luciferase activity, as compared with wild-type MAP1LC3B 3′-UTR (Fig. 5d). Taken together, our data suggested that MAP1LC3B was a novel direct target of miR-342-3p*.*

### Role of tumor-suppressive miR-342-3p and its regulation of DNMT1 and hence methylation-mediated silencing tumor suppressor gene

**Fig. 6:**
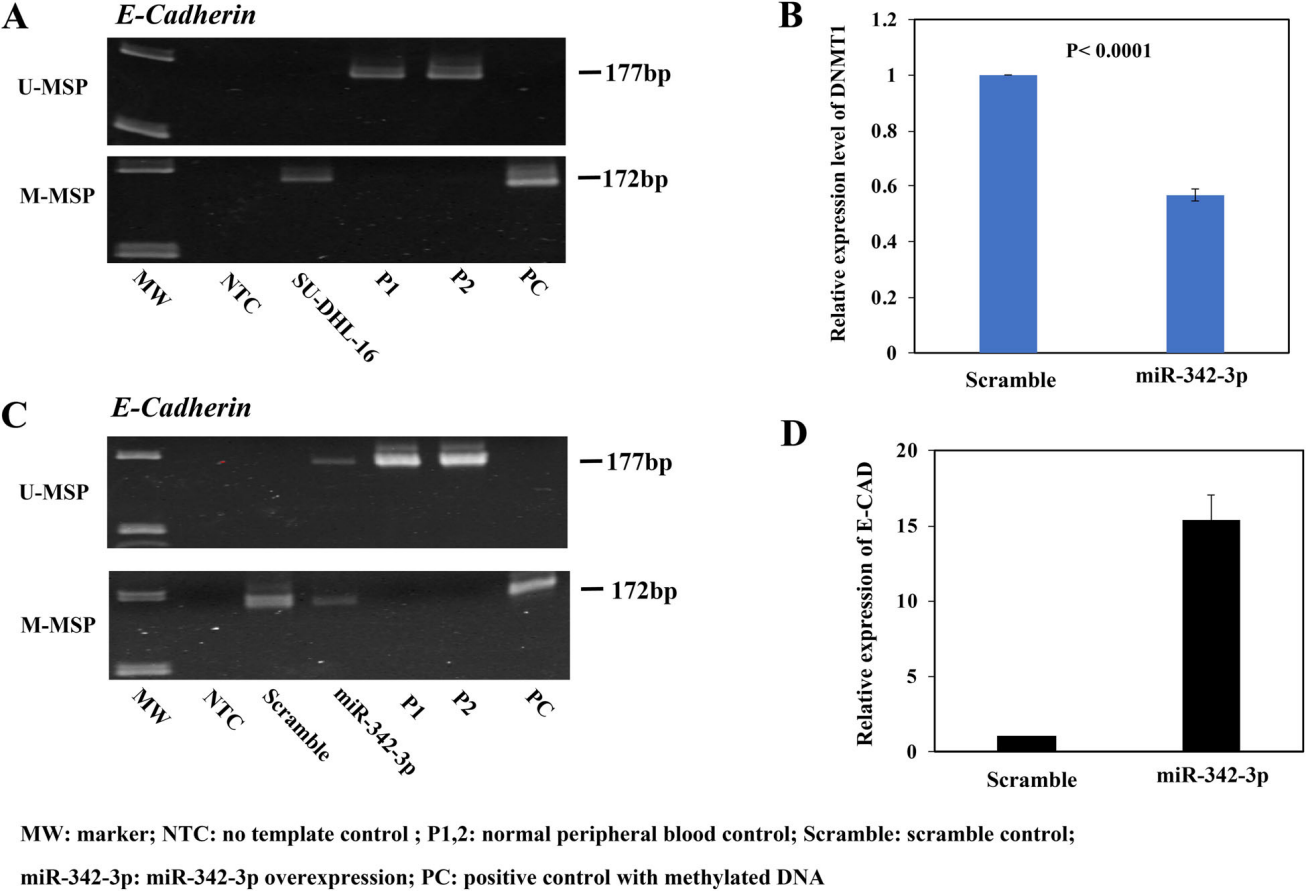
miR-342-3p downregulating DNMT1 and re-expression of E-CAD by promoter demethylation in SU-DHL-16 cells. **a** M- and U-MSP showed *E-CAD* was completely methylated in SU-DHL-16 cells. **b** The expression of DNMT1 decreased upon overexpression of miR-342-3p by qRT-PCR. **c** Effect of overexpression of miR-342-3p on the methylation status of *E-CAD* promoter by MSP. **d** The expression of E-CAD increased upon overexpression of miR-342-3p by qRT-PCR. Data of qRT-PCR were normalized to negative scramble control as 1. Columns represent mean +/− 1SD from three experiments in triplicate

Since miR-342-3p has been shown to target DNMT1 by luciferase reporter assay, and overexpression of miR-342-3p could result in promoter DNA hypomethylation and hence re-expression of hypermethylated tumor suppressor genes in colorectal cancer cells [25], we hypothesized that overexpression of miR-342-3p in completely methylated lymphoma cells would result in downregulation of DNMT1 and hence hypomethylation and re-expression of tumor suppressor genes. Herein, by MSP, E-CAD was shown to be completely methylated in SU-DHL-16 cells (Fig. 6a), which were completely methylated for *EVL/MIR342*. Overexpression of miR-342-3p resulted in downregulation of DNMT1 by qRT-PCR. (P < 0.0001; Fig. 6b). This was associated with promoter DNA demethylation of E-CAD, which was evidenced by the emergence of U-MSP signal (Fig. 6c) and reduction of mean methylation level from 96.57% to 84.57% using quantitative bisulfite pyrosequencing (Additional file 2: Figure S2), and re-expression of E-CAD by qRT-PCR (Fig. 6d).

## Discussion

Herein, there is a number of observations obtained. Firstly, *EVL/MIR342* methylation was detected in NHL cell lines and primary NHL samples, but not in normal peripheral blood and normal tonsil tissues, and hence tumor-specific. The current data were similar to the tumor-specific methylation of other tumor suppressor miRNAs, including *MIR155* [15] and *MIR129-2* [14] in NHL. By contrast, *MIR9-2* [32] and *MIR373* [33] were methylated in both cancer cells and their normal counterparts, hence tissue-specific but not tumor-specific, pathologically irrelevant.

Secondly, our data have demonstrated that the intronic miR-342-3p was co-regulated with its host gene EVL in NHL cells, consistent with data from other studies showing intronic miRNAs, such as miR-28 [34] and miR-3151 [35], which are co-regulated by promoter DNA methylation of their host genes. Therefore, our data have expanded evidence on the view that intronic miRNA is co-regulated by promoter DNA methylation of its host gene [36, 37].

Thirdly, in primary NHL samples, *EVL/MIR342* methylation frequency was differential among different subtypes of NHL with significantly higher frequency in B-cell lymphoma than T-cell lymphoma. Therefore, these results are similar to preferential methylation of *MIR124-1* [17] and *MIR129-2* [14] in B-cell lymphoma. Lymphoma is highly heterogenous with different genetic and epigenetic features [38]. For instance, microarray-based DNA methylation study in 367 hematological neoplasms demonstrated that promoter DNA hypermethylation was more frequent in B and T precursor lymphoid neoplasias as well as mature B-cell lymphomas of germinal center origin such as DLBCL, FL and BL, than mature T-cell lymphomas such as PTCL and AITL [39]. Therefore, differential methylation of *EVL/MIR342* might be accounted by the different cell of origin and hence pathogenetic mechanisms.

Fourthly, our data have shown that the tumor suppressor function of miR-342-3p was mediated by inhibition of pro-survival autophagy in B-cell lymphoma cells. Autophagy is a catabolic process in cellular homeostasis via orderly clearance and recycle of cellular organelles [40]. In tumorigenesis, autophagy is regarded as a “double-edged sword”, whereby tumor suppressive function in cancer initiation and oncogenic function in tumor progression [30, 41]. For instance, while *Becn1* gene is a master player in multiple steps of autophagy [42], *Becn1* mutant mice (*Becn1* ^+/-^) were prone to developing spontaneous lymphoma compared with *Becn1* wild-type mice [43], indicating the tumor suppressor role of autophagy on tumor initiation. In contrast, in SP53 and JEKO-1 MCL cells, repression of autophagy by knockout of *ATG5*, a component of the phagophore elongation complex in autophagy, by lentivirus-mediated CRISPR-Cas9, resulted in inhibition of cellular proliferation and increase of apoptosis [44], suggested the oncogenic role of autophagy for lymphoma. Herein, we have demonstrated the suppression of cellular proliferation and increase of cell death upon pharmacological inhibition of autophagy in B-cell lymphoma cells, thereby consistent with the pro-survival role of autophagy, Moreover, restoration of miR-342-3p led to tumor suppressive effect in B-cell lymphoma cells, but not B-cell lymphoma cells pre-treated with an autophagy inhibitor, 3-MA, thereby confirming a role of pro-survival autophagy in miR-342-3p-mediataed tumor suppressive activity in B-cell lymphoma cells.

Furthermore, our data demonstrated for the first time that inhibition of autophagy by miR-342-3p was mechanistically mediated through direct targeting of MAP1LC3B, which is converted via post-translational modification to LC3-II, a functional marker of autophagy. Therefore, together with our data herein, multiple genes in the autophagy pathway have been shown to be regulated by miRNAs, including targeting of ATG2B by miR-130 in chronic lymphocytic leukemia [45] and ATG14 by miR-140 in multiple myeloma [46]. On the other hand, in contrast to previously reported that miRNA targeting was mediated through the putative SRBS of ≥ 6-mer [47], our data showed that additional two 5-mer binding sites in the 3’UTR of MAP1LC3B contributed to targeting by miR-342-3p, and hence similarly suggesting a role of 5-mer binding sites in miRNAs-mediated posttranscriptional regulation of target genes [48].

Finally, given that DNMT1 is a known target of miR-342-3p [25], herein we showed that over-expression of miR-342-3p led to promoter hypomethylation and re-expression of tumor suppressor gene E-CAD in B-cell lymphoma cells. Interestingly, upregulation of DNMT1 has been shown to correlate with inferior survival in NHL, hence implicated in the pathogenesis of lymphoma [49, 50]. Therefore, methylation-mediated silencing of miR-342-3p leads to upregulation of DNMT1 in B-cell lymphoma with the potential of promoter hypermethylation and repression of multiple tumor suppressor genes.

## Conclusions

In conclusion, our study showed that miR-342-3p is co-regulated with its host gene EVL in NHL by tumor-specific promoter DNA methylation. Moreover, the tumor-suppressive role of miR-342-3p in B cell lymphoma was mediated by inhibition of pro-survival autophagy via direct targeting of MAP1LC3B, a precursor of LC3-II, hence a functional marker of autophagy, in addition to targeting and repression of DNMT1, with consequent hypomethylation and re-expression of tumor suppressor genes.

## Methods

### Patient samples

One hundred and thirty-two preoperatively untreated NHL biopsies at diagnosis from five hospitals in Hong Kong (Queen Mary Hospital, Kwong Wah Hospital, Princess Margaret Hospital, United Christian Hospital and Pamela Youde Nethersole Eastern Hospital), were studied. They included 99 B-cell lymphoma cases and 33 T-cell lymphoma cases. B-cell lymphoma cases comprised 56 DLBCL, 17 mantle cell lymphoma (MCL), six follicular lymphoma (FL), five Burkitt lymphoma (BL), seven small lymphocytic lymphoma (SLL), four mucosa-associated lymphoid tissue lymphoma (MALT), three marginal zone B-cell lymphoma (MZBCL) and one Waldenstrom's macroglobulinemia (WM) case. T-cell lymphoma cases comprised 20 peripheral T-cell lymphoma (PTCL) and 13 angioimmunoblastic T-cell lymphoma (AITL) cases. The diagnosis of NHL was based on the WHO (World Health Organization) classification [38]. Eleven formalin fixed, paraffin-embedded (FFPE) tonsil tissues were obtained from healthy individuals who underwent tonsillectomy. Our study was approved by the Institutional Review Board of Queen Mary Hospital. Samples were collected from patients with informed consent and in accordance with the Declaration of Helsinki.

### Cell culture

Five MCL cell lines (SP53, REC-1, GRANTA-519, MINO, and JEKO-1), two DLBCL cell lines (SU-DHL-6 and SU-DHL-16), two ALK (+) anaplastic large cell lymphoma (ALCL) cell lines (KARPAS-299 and SU-DHL-1), and one T cell lymphoblastic lymphoma cell line (SUP-T1) were included in the current study. SP53 and REC-1 were kindly provided by Prof. Raymond Lai (Department of Laboratory Medicine and Pathology, University of Alberta and Cross Cancer Institute). Other cell lines were purchased from Deutsche Sammlung von Mikroogranismen und Zellkulturen (DSMZ) (Braunschweig, Germany). Cell lines were maintained in RPMI-1640 (DMEM for GRANTA-519) supplemented with 10–15% fetal bovine serum, 50 U/ml of penicillin, and 50 μg/ml streptomycin in a humidified atmosphere of 5% CO_2_ at 37 °C.

### DNA and RNA extraction

Extraction of DNA from NHL cell lines and healthy peripheral blood was performed with DNA Blood Mini kit (Qiagen, Hilden, Germany). Extraction of DNA from NHL patient frozen biopsies was conducted with automated DNA extraction system (DNA Tissue Kit from Qiagen). For FFPE fixed NHL patient samples and normal tonsils, DNA extraction was conducted by using QIAamp DNA FFPE Tissue Kit (Qiagen, Hilden, Germany). Total RNA was extracted with Direct-zol™ RNA MiniPrep kit (Zymo Research).

### Methylation-specific polymerase chain reaction (MSP)

DNA bisulfite treatment was performed to convert unmethylated cytosine into uracil with EpiTect Bisulfite Kit (Qiagen, Hilden, Germany). MSP primers were designed at the CpG island upstream to *MIR342* host gene *EVL* (Additional file 3: Figure S3) and *E-Cadherin (E-CAD)* gene. Details of primer sequence and PCR condition for methylated-MSP (M-MSP) and unmethylated-MSP (U-MSP) were listed in Table 2. The enzymatically methylated control DNA (CpGenome Universal Methylated DNA; Chemicon/Millipore, Billerica, MA, USA) was used as positive control for M-MSP and negative control for U-MSP.

**Table 2:** Primer sequences and PCR reaction conditions for *EVL/MIR342*

	Forward primer (5′ to 3′)	Reverse primer (5′ to 3′)	Tm/cycles/MgCl_**2**_	Reference
(I) **Methylation-specific PCR (MSP)**				
*EVL/MIR342*				
M-MSP	AGG GAG CGT ATC GCG TTA C	CGG AAA ACG CTA AAC TTA AAA CTA CG	55 °C/38x/2 mM	NA
U-MSP	GGG AGG GAG TGT ATT GTG TTA T	CTC CAA AAA CAC TAA ACT TAA AAC TAC A	57 °C/35x/1.5 mM	NA
*E-cadherin*				
M-MSP	GTG GGC GGG TCG TTA GTT TC	CTC ACA AAT ACT TTA CAA TTC CGA CG	62 °C/40x/2 mM	NA
U-MSP	GGT GGG TGG GTT GTT AGT TTT GT	AAC TCA CAA ATA CTT TAC AAT TCC AAC A	58 °C/40x/2 mM	NA
(II) **Quantitative real-time reverse transcription-PCR**				
EVL (NM_001330221.1)	GCA GCG ACA ATG AGT GAA CA	TGC TGA TCC TGC AAC TTG AC		
DNMT1	GCT ACC TGG CTA AAG TCA AA	CCA TTC CCA CTC TAC GG	NA	[30]
E-cadherin	GGA GGA GAG CGG TGG TCA AA	TGT GCA GCT GGC TCA AGT CAA	NA	[31]
GAPDH	ACC ACA GTC CAT GCC ATC ACT	TCC ACC ACC CTG TTG CTG TA	NA	[16]
(III) **Cloning of luciferase reporter constructs with SRBS of miR-342-3p**				
MAP1LC3B	AAG GGC TAG CCC GCC TTT TTG GGT AGA AGT	AGG GTC GAC AGT GAG GAC TTT GGG TGT GG	55 °C/35x/2 mM	NA

*M-MSP* methylated MSP, *U-MSP* unmethylated MSP, *Tm* annealing temperature, *SRBS* seed region binding site

### Quantitative real-time reverse transcription-PCR (qRT-PCR)

Reverse transcription and quantification of miR-342-3p were performed by using TaqMan MicroRNA Reverse Transcription Kit and TaqMan MicroRNA Assay Kit (ABI, Foster City, CA, USA), respectively. RNU48 was used as reference. MAP1LC3B (Cat. Hs00797944_s1) was quantified by Taqman Gene Expression Assay (ABI, Foster City, CA, USA) with GAPDH (Cat. Hs00266705_g1) as endogenous control. For EVL (NM_001330221.1), DNMT1, and E-CAD, RNA was reverse transcribed into cDNA by QuantiTect Reverse Transcription Kit (Qiagen, Hilden, Germany) and quantified by SYBR Green Master Mix (ABI, Foster City, CA, USA). GAPDH was used as the endogenous control. The location of qRT-PCR primers for EVL are shown in Additional file 3: Figure S3. The 2^−ΔΔCt^ method was used to analyze the expression changes of miR-342-3p and EVL before and after 5-AzadC treatment, and the expression changes of miR-342-3p, MAP1LC3B, DNMT1, and E-CAD after transfection of precursor miR-342-3p mimics compared with scrambled oligonucleotides control in SU-DHL-16 cells. Primer sequence for EVL, DNMT1, E-CAD and GAPDH are available in Table 2 [16, 51, 52].

### Quantitative bisulfite pyrosequencing

The promoter regions of *MIR342* host gene *EVL* and *E-CAD*, which overlapped with the amplicon of the MSP, were amplified with methylation-unbiased primers in the bisulfite converted DNA. Primer sequences for pyrosequencing were described previously [53, 54]. For *EVL/MIR342*, forward primer: 5′-GGG GTT AGG AGG GGA TTG GA-3′, reverse primer: 5′-Biotin- TCT CAA CAC AAC AAC CAA AAA CTA-3′; condition: 56 °C/45x/1.5 mM. For *E-CAD*, forward primer: 5′-TTT GAT TTT AGG TTT TAG TGA GT-3′, reverse primer: 5′-Biotin- ACC ACA ACC AAT CAA CAA-3′; condition: 56 °C/45x/2 mM. PyroMark PCR Kit (Qiagen, Hilden, Germany) was employed to perform PCR amplification for pyrosequencing. Thirteen consecutive CpG dinucleotides for *EVL/MIR342* were pyrosequenced with sequencing primer: 5′-AGG AGG GGA TTG GAG GA-3′, and seven consecutive CpG dinucleotides for *E-CAD* were pyrosequenced with sequencing primer: 5′-TAG TAA TTT TAG GTT AGA GG-3′, on a PSQ 96MA system and analyzed using PyroQ-CpG 1.0.9. software. The relative locations of pyrosequencing amplicon, MSP amplicon, and CpG island for *EVL/MIR342* are shown in Additional file 3: Figure S3.

### DNA demethylation treatment

SU-DHL-16 cells were seeded at a density of 1 × 10^6^ cells/ml in 25 cm^2^ flasks and cultured with 0.5–1.5 μM of 5-AzadC (Sigma-Aldrich, St. Louis, MO) for 6 days, with fresh medium and 5-AzadC replaced at every 24 h. Cells were harvested on day 6 for DNA and RNA extraction.

### Transfection of precursor miR-342-3p mimics

For cellular proliferation, viability assays, and Western-blotting, SU-DHL-16 cells (1 × 10^6^ cells/ml) were transfected with either precursor miR-342-3p mimics or negative scramble oligonucleotides control (Ambion, Austin, TX, USA) at final concentration of 100 nM with Lipofectamine 2000 transfection reagent (Invitrogen, Carlsbad, CA, USA) according to manufacturer’s instructions. The transfected cells were cultured under serum-free conditions for 120 h. For study of miR-342-3p targeting DNMT1, SU-DHL-16 (0.5 × 10^6^ cells/ml) were transfected with either 150 nM precursor miR-342-3p mimics or scramble control for two consecutive days. After 24 h of second transfection, the transfection medium was substituted with RPMI-1640 containing 10% FBS and continued to culture for 24–72 h.

### MTS assay and trypan blue staining

Cellular proliferation was measured by MTS assay with CellTiter 96® AQ_ueous_ One Solution Cell Proliferation Assay kit (Promega, USA). Briefly, transfected cells were seeded into a 96-well plate at the density of 2.5 × 10^4^ cells/well in 100 μl medium. At 120 h post-transfection, 20 μl MTS reagent was added into each well and incubated for 4 h. Then the absorbance reading at 490 nm was recorded. Percentage of dead cells was determined by trypan blue dye exclusion assay under microscope at 120 h after transfection. Both dead cells and viable cells in five random microscopic fields were counted. Percentage of dead cells was calculated as: (total number of dead cells per microscopic field/total number of dead cells and viable cells per microscopic field) × 100%. Each assay was repeated in triplicate from three independent transfections.

### Western-blotting

SU-DHL-16 cells transfected with either precursor miR-342-3p mimics or scramble control were harvested at 120 h post-transfection and lysed in RIPA buffer (Cell Signaling Technology, Danvers, MA, USA) with protease inhibitors and phosphatase inhibitor Cocktail (Cell Signaling Technology). Cell lysates containing 10 μg protein were separated on Mini-PROTEAN TGX™ 10% SDS-PAGE gel (Bio-Rad, Hercules, CA, USA) and transferred to a 0.45-μm PVDF membrane (GE Healthcare, Chicago, IL, USA). The membranes were blocked and then incubated with primary antibodies (Rabbit anti-human, anti-LC3A/B 1:1000, anti-actin 1:5000; Cell Signaling Technology) at 4 °C overnight. Thereafter, membranes were washed and incubated with secondary antibody (Anti-rabbit IgG, HRP-linked Antibody 1:3000, Cell Signaling) for 1 h at room temperature with gently shaking, followed by detection of protein signals with X-ray film. The integral optical density of protein bands was quantified by Image J software.

### Target prediction analysis

Putative autophagy-related targets of miR-342-3p, matching to the 6-mer, 7mer-A1, 7mer-m8 and 8-mer miRNA seed sequences [47], were predicted using miRWalk2.0 bioinformatic program [31], a bioinformatics platform comprising several common miRNA target prediction databases, including Microt4, miRanda, miRDB, TargetScan, RNA22 and PITA.

### Plasmid constructs

A 3′-UTR DNA segment of MAP1LC3B (~ 230 bp) containing putative seed region binding site (SRBS; Position: 536-545nts of 3′-UTR) of miR-342-3p was amplified and cloned into the *Nhe*I and *Sal*I sites of a dual firefly/renilla luciferase reporter vector, pmirGLO (Promega). MAP1LC3B 3′-UTR mutants with deletion of putative miR-342-3p binding sites were synthesized as gBlocks Gene Fragments (Integrated DNA Technologies, Coralville, IA, USA) and cloned into the pmirGLO (Promega). The sequence of PCR primers and PCR condition are summarized in Table 2.

### Luciferase reporter assay

HeLa cells (kindly provided by Dr Zou, Department of Medicine, The University of Hong Kong) were co-transfected with 500 ng wild-type/mutant plasmids and precursor miR-342-3p mimics/negative scramble control at a final concentration of 50 nM with Lipofectamine 2000 transfection reagent in a 24-well plate. At 48 h post-transfection, transfected cells were harvested. The luminescent signal was quantified by Dual-Luciferase Reporter Assay System (Promega) by CLARIOstar (BMG Labtech). Firefly luciferase activity was normalized by Renilla luciferase activity. Each experiment was conducted in triplicate from three independent transfections.

### Statistical analysis

The mean expression of miR-342-3p, EVL, or MAP1LC3B between methylated and unmethylated NHL cell lines or patient samples were compared by the Student’s *t* test. The difference of trypan blue exclusion assay and MTS assay between SU-DHL-16 cells transfected with miR-342-3p mimics and scrambled oligonucleotides control were compared by Student’s *t* test. The difference of *EVL/MIR342* methylation frequency in different subtypes of NHL primary samples was analyzed by *χ*^2^ test. Correlation between *EVL/MIR342* methylation with age and gender were studied by Student’s *t* test and *χ*^2^ test, respectively. Overall survival was plotted by the Kaplan-Meier method and compared by the log-rank test. All *P* values were 2-sided. *P* < 0.05 was considered as significant difference.

## Supplementary information

**Additional file 1: Figure S1**. Quantitative pyrosequencing analysis interrogating mean methylation percentage over a stretch of 13 neighboring CpG dinucleotides embedded in the promoter-associated CpG island of *EVL/MIR342* in 0% normal control, 50% methylation control, 100% methylation positive control, and NHL cell lines.

**Additional file 2: Figure S2**. Effect of overexpression of miR-342-3p on the methylation status of *E-CAD* promoter by quantitative bisulfite pyrosequencing.

**Additional file 3: Figure S3**. Schematic diagram showing the relative locations of *MIR342* (red)*, EVL* gene, CpG island (green), amplicon of methylation specific PCR (MSP) (blue) and bisulfite pyrosequencing (yellow).

## Data Availability

All data generated or analyzed during this study are included in this published article.

## Abbreviations

NHL: Non-Hodgkin’s lymphoma
MSP: Methylation-specific PCR
TSG: Tumor suppressive gene
miRNA: MicroRNA
3′-UTR: 3′-Untranslated region
EVL: Enah/Vasp-like
DLBCL: Diffuse large B cell lymphoma
MCL: Mantle cell lymphoma
FL: Follicular lymphoma
BL: Burkitt’s lymphoma
SLL: Small lymphocytic lymphoma
MALT: Mucosa-associated lymphoid tissue lymphoma
MZBCL: Marginal zone B cell lymphoma
WM: Waldenstrom’s macroglobulinemia
PTCL: Peripheral T cell lymphoma
AITL: Angioimmunoblastic T cell lymphoma
ALCL: Anaplastic large cell lymphoma
FFPE: Formalin fixed, paraffin-embedded
M-MSP: Methylated-MSP
U-MSP: Unmethylated-MSP
qRT-PCR: Quantitative real-time reverse transcription-PCR
5-AzadC: 5-Aza-2′-deoxycytidine
MM: Completely methylated
MU: Partially methylated
UU: Completely unmethylated
MAP1LC3B: Microtubule-associated proteins 1A/1B light chain 3B
SRBS: Seed region binding site
3-MA: 3-Methyladenine
ATG: Autophagy-related gene
E-CAD: E-Cadherin

## References

[1] Bray F, Ferlay J, Soerjomataram I, Siegel RL, Torre LA, Jemal A (2018). Global cancer statistics 2018: GLOBOCAN estimates of incidence and mortality worldwide for 36 cancers in 185 countries. CA Cancer J Clin.

[2] Yim RL, Kwong YL, Wong KY, Chim CS (2012). DNA methylation of tumor suppressive miRNAs in non-Hodgkin's lymphomas. Front Genet.

[3] Sun J, Yang Q, Lu Z, He M, Gao L, Zhu M, Sun L (2012). Distribution of lymphoid neoplasms in China: analysis of 4,638 cases according to the World Health Organization classification. Am J Clin Pathol.

[4] Chim CS, Ma SY, Au WY, Choy C, Lie AK, Liang R, Yau CC (2004). Primary nasal natural killer cell lymphoma: long-term treatment outcome and relationship with the International Prognostic Index. Blood..

[5] Esteller M (2008). Epigenetics in cancer. N Engl J Med.

[6] Klutstein M, Nejman D, Greenfield R, Cedar H (2016). DNA methylation in cancer and aging. Cancer Res.

[7] Chim CS, Wong KY, Loong F, Lam WW, Srivastava G (2007). Frequent epigenetic inactivation of Rb1 in addition to p15 and p16 in mantle cell and follicular lymphoma. Hum Pathol.

[8] Chim CS, Wong KY, Loong F, Srivastava G (2004). SOCS1 and SHP1 hypermethylation in mantle cell lymphoma and follicular lymphoma: implications for epigenetic activation of the Jak/STAT pathway. Leukemia..

[9] Molavi O, Wang P, Zak Z, Gelebart P, Belch A, Lai R (2013). Gene methylation and silencing of SOCS3 in mantle cell lymphoma. Br J Haematol.

[10] Lopez-Serra P, Esteller M (2012). DNA methylation-associated silencing of tumor-suppressor microRNAs in cancer. Oncogene..

[11] Esquela-Kerscher A, Slack FJ (2006). Oncomirs - microRNAs with a role in cancer. Nat Rev Cancer.

[12] Bartel DP (2009). MicroRNAs: target recognition and regulatory functions. Cell..

[13] Calin GA, Croce CM (2006). MicroRNA signatures in human cancers. Nat Rev Cancer.

[14] Wong KY, Yim RL, Kwong YL, Leung CY, Hui PK, Cheung F, Liang R (2013). Epigenetic inactivation of the MIR129-2 in hematological malignancies. J Hematol Oncol.

[15] Yim RL, Wong KY, Kwong YL, Loong F, Leung CY, Chu R, Lam WW (2014). Methylation of miR-155-3p in mantle cell lymphoma and other non-Hodgkin's lymphomas. Oncotarget..

[16] Chim CS, Wong KY, Qi Y, Loong F, Lam WL, Wong LG, Jin DY (2010). Epigenetic inactivation of the miR-34a in hematological malignancies. Carcinogenesis..

[17] Wong KY, So CC, Loong F, Chung LP, Lam WW, Liang R, Li GK (2011). Epigenetic inactivation of the miR-124-1 in haematological malignancies. PLoS One.

[18] Wilton KM, Overlee BL, Billadeau DD (2019). NKG2D-DAP10 signaling recruits EVL to the cytotoxic synapse to generate F-actin and promote NK cell cytotoxicity. J Cell Sci.

[19] Krause M, Dent EW, Bear JE, Loureiro JJ, Gertler FB (2003). Ena/VASP proteins: regulators of the actin cytoskeleton and cell migration. Annu Rev Cell Dev Biol.

[20] Kwiatkowski AV, Gertler FB, Loureiro JJ (2003). Function and regulation of Ena/VASP proteins. Trends Cell Biol.

[21] Hu LD, Zou HF, Zhan SX, Cao KM (2008). EVL (Ena/VASP-like) expression is up-regulated in human breast cancer and its relative expression level is correlated with clinical stages. Oncol Rep.

[22] Fathallah-Shaykh HM, Rigen M, Zhao LJ, Bansal K, He B, Engelhard HH, Cerullo L (2002). Mathematical modeling of noise and discovery of genetic expression classes in gliomas. Oncogene..

[23] Li XR, Chu HJ, Lv T, Wang L, Kong SF, Dai SZ (2014). miR-342-3p suppresses proliferation, migration and invasion by targeting FOXM1 in human cervical cancer. FEBS Lett.

[24] Grady WM, Parkin RK, Mitchell PS, Lee JH, Kim YH, Tsuchiya KD, Washington MK (2008). Epigenetic silencing of the intronic microRNA hsa-miR-342 and its host gene EVL in colorectal cancer. Oncogene..

[25] Wang H, Wu J, Meng X, Ying X, Zuo Y, Liu R, Pan Z (2011). MicroRNA-342 inhibits colorectal cancer cell proliferation and invasion by directly targeting DNA methyltransferase 1. Carcinogenesis..

[26] Zhao L, Zhang Y (2015). miR-342-3p affects hepatocellular carcinoma cell proliferation via regulating NF-kappaB pathway. Biochem Biophys Res Commun.

[27] Tai MC, Kajino T, Nakatochi M, Arima C, Shimada Y, Suzuki M, Miyoshi H (2015). miR-342-3p regulates MYC transcriptional activity via direct repression of E2F1 in human lung cancer. Carcinogenesis..

[28] Crippa E, Folini M, Pennati M, Zaffaroni N, Pierotti MA, Gariboldi M (2016). miR-342 overexpression results in a synthetic lethal phenotype in BRCA1-mutant HCC1937 breast cancer cells. Oncotarget..

[29] Weng W, Okugawa Y, Toden S, Toiyama Y, Kusunoki M, Goel A (2016). FOXM1 and FOXQ1 are promising prognostic biomarkers and novel targets of tumor-suppressive miR-342 in human colorectal cancer. Clin Cancer Res.

[30] Levy JMM, Towers CG, Thorburn A (2017). Targeting autophagy in cancer. Nat Rev Cancer.

[31] Dweep H, Gretz N (2015). miRWalk2.0: a comprehensive atlas of microRNA-target interactions. Nat Methods.

[32] Wang LQ, Kwong YL, Kho CS, Wong KF, Wong KY, Ferracin M, Calin GA (2013). Epigenetic inactivation of miR-9 family microRNAs in chronic lymphocytic leukemia--implications on constitutive activation of NFkappaB pathway. Mol Cancer.

[33] Lujambio A, Esteller M (2007). CpG island hypermethylation of tumor suppressor microRNAs in human cancer. Cell Cycle.

[34] Li Z, Wong KY, Chan GC, Chim CS (2018). Epigenetic silencing of LPP/miR-28 in multiple myeloma. J Clin Pathol.

[35] Wang LQ, Wong KY, Rosen A, Chim CS (2015). Epigenetic silencing of tumor suppressor miR-3151 contributes to Chinese chronic lymphocytic leukemia by constitutive activation of MADD/ERK and PIK3R2/AKT signaling pathways. Oncotarget..

[36] Rodriguez A, Griffiths-Jones S, Ashurst JL, Bradley A (2004). Identification of mammalian microRNA host genes and transcription units. Genome Res.

[37] Baskerville S, Bartel DP (2005). Microarray profiling of microRNAs reveals frequent coexpression with neighboring miRNAs and host genes. Rna..

[38] Swerdlow SH, Campo E, Harris NL, Jaffe ES, Pileri SA, Stein H, Thiele J (2008). WHO classification of tumours of haematopoietic and lymphoid tissues.

[39] Martin-Subero JI, Ammerpohl O, Bibikova M, Wickham-Garcia E, Agirre X, Alvarez S, Bruggemann M (2009). A comprehensive microarray-based DNA methylation study of 367 hematological neoplasms. PLoS One.

[40] Choi AM, Ryter SW, Levine B (2013). Autophagy in human health and disease. N Engl J Med.

[41] Kimmelman AC, White E (2017). Autophagy and tumor metabolism. Cell Metab.

[42] Kang R, Zeh HJ, Lotze MT, Tang D (2011). The Beclin 1 network regulates autophagy and apoptosis. Cell Death Differ.

[43] Yue Z, Jin S, Yang C, Levine AJ, Heintz N (2003). Beclin 1, an autophagy gene essential for early embryonic development, is a haploinsufficient tumor suppressor. Proc Natl Acad Sci U S A.

[44] Zhang H, Chen Z, Miranda RN, Medeiros LJ, McCarty N (2016). TG2 and NF-kappaB signaling coordinates the survival of mantle cell lymphoma cells via IL6-mediated autophagy. Cancer Res.

[45] Kovaleva V, Mora R, Park YJ, Plass C, Chiramel AI, Bartenschlager R, Dohner H (2012). miRNA-130a targets ATG2B and DICER1 to inhibit autophagy and trigger killing of chronic lymphocytic leukemia cells. Cancer Res.

[46] Lu D, Yang C, Zhang Z, Cong Y, Xiao M (2018). Knockdown of Linc00515 inhibits multiple myeloma autophagy and chemoresistance by upregulating miR-140-5p and downregulating ATG14. Cell Physiol Biochem.

[47] Grimson A, Farh KK, Johnston WK, Garrett-Engele P, Lim LP, Bartel DP (2007). MicroRNA targeting specificity in mammals: determinants beyond seed pairing. Mol Cell.

[48] Hart M, Kern F, Backes C, Rheinheimer S, Fehlmann T, Keller A, Meese E (2018). The deterministic role of 5-mers in microRNA-gene targeting. RNA Biol.

[49] Zhao H, Zhang LE, Guo S, Yuan T, Xia B, Zhang L, Zhang Y (2015). Overexpression of DNA methyltransferase 1 as a negative independent prognostic factor in primary gastrointestinal diffuse large B-cell lymphoma treated with CHOP-like regimen and rituximab. Oncol Lett.

[50] Robaina MC, Mazzoccoli L, Arruda VO, Reis FR, Apa AG, de Rezende LM, Klumb CE (2015). Deregulation of DNMT1, DNMT3B and miR-29s in Burkitt lymphoma suggests novel contribution for disease pathogenesis. Exp Mol Pathol.

[51] Huang J, Wang Y, Guo Y, Sun S (2010). Down-regulated microRNA-152 induces aberrant DNA methylation in hepatitis B virus-related hepatocellular carcinoma by targeting DNA methyltransferase 1. Hepatology..

[52] Cao Q, Yu J, Dhanasekaran SM, Kim JH, Mani RS, Tomlins SA, Mehra R (2008). Repression of E-cadherin by the polycomb group protein EZH2 in cancer. Oncogene..

[53] Kaz AM, Wong CJ, Dzieciatkowski S, Luo Y, Schoen RE, Grady WM (2014). Patterns of DNA methylation in the normal colon vary by anatomical location, gender, and age. Epigenetics..

[54] Rusiecki JA, Al-Nabhani M, Tarantini L, Chen L, Baccarelli A, Al-Moundhri MS (2011). Global DNA methylation and tumor suppressor gene promoter methylation and gastric cancer risk in an Omani Arab population. Epigenomics..

