# Supplementary figures and images for: Epigenetic silencing of miR-342-3p in B cell lymphoma and its impact on autophagy
Source: Clin Epigenetics. 2020 Oct 19;12:150. doi: 10.1186/s13148-020-00926-1 (PMC7574348; doi:10.1186/s13148-020-00926-1)

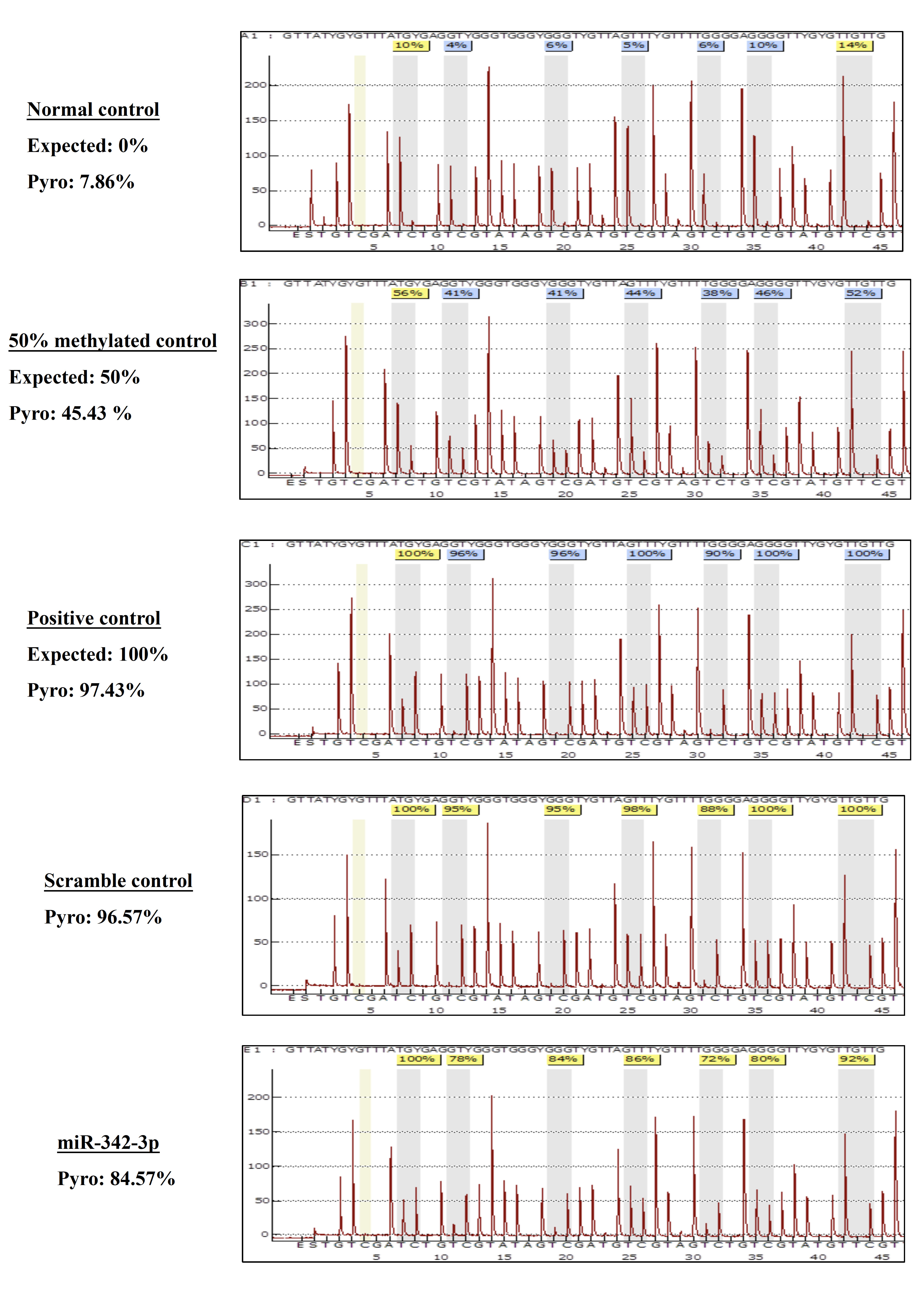

Supplement: Supplementary file 2 — Additional file 2: Figure S2. Effect of overexpression of miR-342-3p on the methylation status of E-CAD promoter by quantitative bisulfite pyrosequencing. [file 13148_2020_926_MOESM2_ESM.jpg]

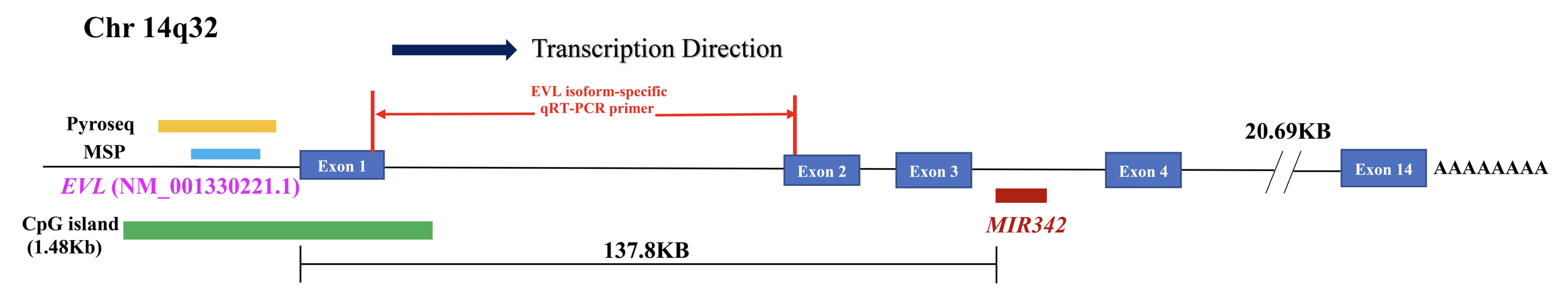

Supplement: Supplementary file 3 — Additional file 3: Figure S3. Schematic diagram showing the relative locations of MIR342 (red), EVL gene, CpG island (green), amplicon of methylation specific PCR (MSP) (blue) and bisulfite pyrosequencing (yellow). [file 13148_2020_926_MOESM3_ESM.jpg]
